# Minimum requirements for the estimation of measurement uncertainty: Recommendations of the joint Working group for uncertainty of measurement of the CSMBLM and CCMB

**DOI:** 10.11613/BM.2017.030502

**Published:** 2017-10-15

**Authors:** Ivana Ćelap, Ines Vukasović, Gordana Juričić, Ana-Maria Šimundić

**Affiliations:** 1 CSMBLM, Committee for the scientific professional development, Working group for uncertainty of measurement of the CSMBLM and CCMB, Croatia; 2 Department of Clinical Chemistry, Sestre milosrdnice University Hospital Center, Zagreb, Croatia; 3 Department of Laboratory Diagnostics, General Hospital Pula, Pula, Croatia; 4 Department of Medical Laboratory Diagnostics, Clinical Hospital Sveti Duh, Zagreb, Croatia

**Keywords:** measurement uncertainty, recommendations, harmonization, analytical phase

## Abstract

The International vocabulary of metrology – Basic and general concepts and associated terms (VIM3, 2.26 measurement uncertainty, JCGM 200:2012) defines uncertainty of measurement as a non-negative parameter characterizing the dispersion of the quantity values being attributed to a measurand, based on the information obtained from performing the measurement. Clinical Laboratory Standards Institute (CLSI) has published a very detailed guideline with a description of sources contributing to measurement uncertainty as well as different approaches for the calculation (Expression of measurement uncertainty in laboratory medicine; Approved Guideline, CLSI C51-A 2012). Many other national and international recommendations and original scientific papers about measurement uncertainty estimation have been published. In Croatia, the estimation of measurement uncertainty is obligatory for accredited medical laboratories. However, since national recommendations are currently not available, each of these laboratories uses a different approach in measurement uncertainty estimation. The main purpose of this document is to describe the minimal requirements for measurement uncertainty estimation. In such way, it will contribute to the harmonization of measurement uncertainty estimation, evaluation and reporting across laboratories in Croatia. This recommendation is issued by the joint Working group for uncertainty of measurement of the Croatian Society for Medical Biochemistry and Laboratory Medicine and Croatian Chamber of Medical Biochemists. The document is based mainly on the recommendations of Australasian Association of Clinical Biochemists (AACB) Uncertainty of Measurement Working Group and is intended for all medical biochemistry laboratories in Croatia.

## Introduction

Measurement uncertainty estimation results from the need for comparison of laboratory results. According to EN ISO 15189 (3.17.) uncertainty of measurement is “a parameter associated with the result of a measurement that characterises the dispersion of the values that could reasonably be attributed to the measurand“ (*1*). It means that measurement uncertainty gives us the range of values where we could expect a true value of the measurand (or a quantifiable property of the analyte) with the same probability. Further, one should not consider measurement uncertainty as an indicator of the measurement system error but rather as a result of the variability of the measurement conditions. Thus, it is the property of the measurement result.

In the analytical process, the source of uncertainty could be any process, which contributes to uncertainty of measurement result. The sources of uncertainty can be found in the preanalytical, analytical and postanalytical phase. Unfortunately, the sources of uncertainty in the preanalytical and postanalytical phase cannot be quantified. Thus, the estimation of measurement uncertainty can be done only for the analytical phase.

Measurement uncertainty may be expressed as standard, relative, combined and expanded (*2*). Opposed to analytical chemistry laboratories, which always present measurement result with its uncertainty, in medical laboratories the estimated measurement uncertainty is not commonly expressed with measurement result on a laboratory report. However, the information on measurement uncertainty should be easily accessible to the users of laboratory services on demand.

Estimation of measurement uncertainty and its periodical verification ensure that a laboratory meets defined quality specifications of the methods, and offers the possibility to evaluate significant differences between two measurements.

To date, there are over twenty international guidelines issued by national standardization institutes, professional associations and accreditation bodies, which explain methods for the estimation and expression of measurement uncertainty (*2*-*13*). Unfortunately, all of these guidelines propose different approaches in measurement uncertainty estimation, different components for uncertainty budget and different equations in measurement uncertainty calculation. There is no agreement between experts on international level on how to estimate and express the measurement uncertainty. Most of the published guidelines include coefficient of variation of repeated measurements, bias and uncertainty of calibrator.

However, in laboratories the data on bias and uncertainty of calibrator are often not known or not easily available. The main reason for that is lack of use of certified reference materials (CRM) or primary standards. The quantity of the measurand in CRM is measured using a reference method calibrated to the primary standard. That is why CRM is used for bias estimation. Furthermore, if one would like to input uncertainty of calibrator in the uncertainty budget, that information should be available. However, in user specifications of calibrators, manufacturers do not provide information on uncertainty of calibrator for the particular lot (*14*). Laboratories could obtain that information from the manufacturers on demand. Nevertheless, it should be emphasized that obtained data on uncertainty of calibrator is not lot specific. Since significant deviations between lots of the same calibrator could be noticed, it is clear that uncertainty of calibrator could not be the same for each lot. Thus, each series of calibrator should be accompanied with the information on its uncertainty.

**Figure 1:**
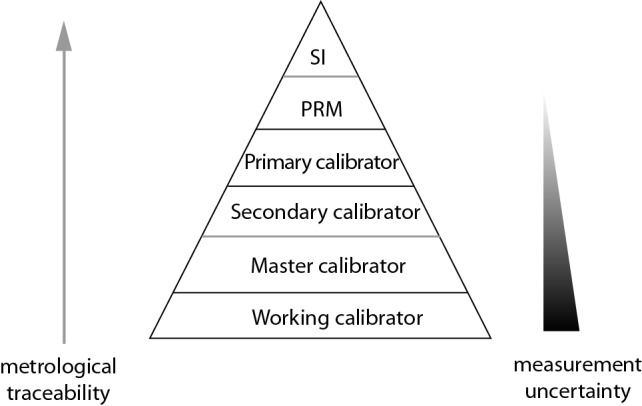
Metrological traceability – property of a measured result whereby the result can be related to the reference through a documented unbroken chain of calibrators, each contributing to the measurement uncertainty (*18*). SI – International System of Units – unit of measurement - scalar quantity which is conventionally defined and adopted with which every other quantity of the same kind can be compared to express the ratio of the second quantity to the first one as a number (*18*). PRM – primary reference material. Primary and secondary calibrators are the calibrators of the highest level in hierarchy and their uncertainty is the lowest in relation to the primary reference material. Lower level calibrators are made according to primary and secondary calibrators. Master calibrator is a lower level calibrator in hierarchy and is used for working calibrator production by manufacturers. Working calibrators are used in routine medical laboratories.

Unfortunately, according to IVD Directive 98/79/EC, information on uncertainty of calibrator for each lot is not obligatory for the manufacturer (*15*). Moreover, only a few manufacturers express uncertainty of calibrator according to primary standard (or primary reference material) while most of them express it as an uncertainty according to master calibrator (Figure 1). It can be concluded that even if the manufacturer provides the information on calibrator uncertainty we do not know according to which higher standard is that uncertainty expressed.

Although EU Regulation 2017/746 (effective from May 5^th^, 2017) obliges manufacturers to provide information according to which higher standard is uncertainty of the calibrator expressed, full implementation of the Regulation is extended by 2022 (*16*).

In order to be compliant with the international recommendations for measurement uncertainty estimation, with respect to the above mentioned problems in trueness detection and uncertainty of calibrator, it is this working group’s opinion that uncertainty of measurement could be reliably estimated from the internal quality data gained through appropriate period of time. The main presumption is that the control sample is commutable and the measurand has the same property as in the patient sample, respectively. In addition, if we are not sure about the commutability of the control sample, patient sample can be regarded as a convenient replacement with the condition that the measured value is near some point of interest (cut-off value or clinical decision limit values).

The period chosen for the data analysis should be long enough to cover a respectable number of changes of reagents, calibrators, control samples, analyser maintenances, working procedures, staff included in these procedures and environmental conditions.

This recommendation was done by the joint Working group for uncertainty of measurement of the Croatian Society of Medical Biochemistry and Laboratory Medicine (CSMBLM) and Croatian Chamber of Medical Biochemists (CCMB) based on available literature searched through the web sites of the international societies of laboratory medicine, *i.e.* the Australasian Association of Clinical Biochemists (AACB), The Royal College of Pathologists of Australasia (RCPA), the International Federation for Clinical Chemistry and Laboratory Medicine (IFCC); standardization institutes, *i.e.* State Office for Metrology (BIPM), Clinical and Laboratory Standards Institute (CLSI); accreditation bodies (Ontario Laboratory Accreditation, American Association for Laboratory Accreditation (A2LA), Clinical Pathology Accreditation (CPA UK), National Pathology Accreditation Advisory Council (NPAAC), Singapore Accreditation Council (SAC) and published papers (*2*-*11*, *17*-*26*).

Using our own data, we evaluated all the suggested approaches in measurement uncertainty estimation, and concluded that the most appropriate approach in laboratory medicine could be the one suggested by White *et al.* which is supported by Burnett and Westgard (*17*, *24*-*27*). This approach ensures that every medical laboratory could estimate measurement uncertainty using its own data with minimal employee effort and without burdening financial budget.

This recommendation gives instructions on how to estimate uncertainty of measurement of quantitative tests using CCMB recommended methods. The recommendation is intended to all medical biochemistry laboratories in Croatia, regardless of the level of health care they provide or accreditation status. In such way, it will contribute to the uniformity of the measurement uncertainty estimation and improvement of the test results comparability on the national level.

## How to use the estimated measurement uncertainty

The estimation of measurement uncertainty has its practical use in evaluation of laboratory test results. It is of utmost importance at the clinical outcome cut-off levels, reference interval limits and significance of a difference between two measurements. Further, estimated measurement uncertainty obtained from long-term internal quality control (IQC) data is used for periodical assessment of the analytical quality specifications set by laboratory.

By knowing the measurement uncertainty (U), a specialist in laboratory medicine can correctly perceive measurement result and provide reliable patient care and safety. Therefore, it is of utmost importance that the measurement uncertainty estimation is performed at the clinical decision limits or cut-off values. Following, the 1st Strategic Conference of the European Federation of Clinical Chemistry and Laboratory Medicine (EFLM) held in Milan, defined a hierarchy of models which are to be used to set analytical performance specifications (*28*). The first model is based on the effect of analytical performance on clinical outcomes, the second is based on components of biological variation (BV) of the measurands, and the third model is based on state-of-the-art which relates to the highest level of analytical performance technically achievable. According to EFLM Task and Finish Group on Allocation of laboratory tests to different models for performance specifications (TFG-DM), the first model can be applied on measurands which have central role in decision making and with established cut-off or decision limits (lipids, plasma glucose, albumin or troponins, *etc.*) (*29*). The BV model can be applied to most measurands but with limitation and need to re-evaluate the validity of BV data (*28*-*30*). This model can be applied on measurands under strict homeostatic control and stable concentrations, or with measurands where deviation from its stable concentration will not cause symptoms (plasma electrolytes and minerals, creatinine, urea, urate, haemoglobin, *etc*.). The state-of-the-art model is applied on measurands, which cannot be included in the two previously described models, and it covers mainly the measurands in urine. The significance of the result of the measurement uncertainty estimation can be assessed by comparing the result with the specifications defined according to models proposed in the Milan conference, or with the total allowable error (TEa) based on biological variation components as proposed by some authors (*31*-*33*). Further, we can use the criteria defined by some expert groups, or the criteria specified by manufacturers. The source of the criteria does not have to be the same for all laboratory tests but can be set based on available literature data and depending on the clinical use of the test (diagnostic, prognostic, monitoring).

Information on measurement uncertainty could produce corrective actions related to improvement of analytical quality of the method. For example, laboratory can replace a calibrator in use with a new one with lower measurement uncertainty; ensure long-term use of the same lot of reagents or more frequently calibrate the analyser. If the analytical quality specifications cannot be obtained, despite corrective actions, laboratory can initiate replacement of the current method with the better one if such is available at the market.

The opinion of this working group is that the coefficient of variation gained through laboratories´ internal quality control data is sufficient for satisfying the minimal criteria for measurement uncertainty estimation. The influences of other uncertainties, such as those of pipettes used to dissolve calibrator or control material, or uncertainty of calibrator material is also reflected through the quality control results *i.e.* coefficient of variation, so they are not separately taken into account when estimating measurement uncertainty. Because of the reasons mentioned earlier about the CRM availability, this working group will not obligate laboratories to introduce bias into measurement uncertainty estimation. However, it is advisable for laboratories, which possess CRMs, to include bias into the equation for uncertainty of measurement estimation.

As for the criteria limits, since the first model from Milan Conference requires clinical outcome studies which are still lacking, we recommend the use of quality specifications data for imprecision or, if bias is taken into account, for total error (TE) from one of the biological variation databases specification (Ricos or other freely available databases). Namely, if our measurement uncertainty (U) is based only on coefficient of variation and is expressed with coverage factor of k = 2, then the acceptance criteria is 2 x imprecision (I, %) (Appendix 1, Example 1). If we have a CRM and take bias into account, then the acceptance criteria is total error (TE, %) (Appendix 1, Example 2).

If the estimated measurement uncertainty does not meet the goal set, laboratory should analyse sources of variability within the measurement process and implement the bottom up approach for measurement uncertainty estimation.

## Measurement uncertainty estimation from verification data

Before the implementation of a method into routine practice, measurement uncertainty should be estimated from the verification procedure data. The method performance verification includes precision data obtained according to CLSI EP15-A2 protocol (*34*).

Quantity of the interest is measured in commercial control samples in triplicate, in five consecutive days. Repeatability (within run precision), reproducibility (between run precision) and within-laboratory precision (total laboratory precision) are calculated from the obtained results with equations presented in Appendix 2 (*i.e.* Eq. 5, Eq. 7, Eq. 9, respectively).

Within-laboratory precision represents standard measurement uncertainty (u). If expressed as coefficient of variation, within-laboratory precision represents relative standard measurement uncertainty (u_rel_) (*2*). Depending on the desired level of confidence, the appropriate coverage factor (k) should be applied to give an expanded uncertainty (U). For an approximately 95% level of confidence k is 2. The equation for expanded relative measurement uncertainty (U_rel_) is: U_rel_ = u_rel_ x 2 (*2*).

## Measurement uncertainty estimation from long-term IQC data

After a certain period of the method routine use and when sufficient number of IQC data is collected (*i.e.* 6 months) measurement uncertainty should be estimated again. Measurement uncertainty should be estimated after and every 6 months of the routine use for methods where IQC is carried out daily. If IQC is carried out less frequently than measurement uncertainty should be estimated every 12 months.

Every laboratory should implement their own IQC frequency depending on the number of samples and/or batches and IQC policy. For example, for a small number of samples per batch IQC could be carried out once a day and for large number of samples and batches IQC could be carried out several times per day (*35*).

Further, every laboratory should pay attention to the selection of control samples, their storage and manipulation. To avoid variability in control material preparation it is advisable to use ready-to-use control materials. Lyophilised control materials should be carefully prepared since the measurement uncertainty of the pipette could contribute to variability of IQC data. Thus, pipettes should be regularly calibrated. Further critical parameters are: aliquoting, type of container, storage conditions, sample freezing, homogeneity of thawed sample and manipulation with the sample according to manufacturers´ recommendations.

In cases where manipulation with the control sample differs from patient sample manipulation, estimation of measurement uncertainty requires careful investigation and, if needed, inclusion of other sources of variability (*e.g.* pipette).

Laboratory should estimate yearly supply of the commercial control samples as a base for tenders to arrange sufficient quantity of the control material with the same target value or the same production series (at least 6 months).

The long-term coefficient of variation represents u_rel_, which multiplied with coverage factor gives U_rel_. Measurement result can be expressed together with its measurement uncertainty expressed as:

- percentage (measured value ± U_rel_)
- absolute value expressed in measurement unit (measured value ± U) (*2*).

Expanded measurement uncertainty (k = 2) represents 95% confidence interval (95% CI) of measured results.

If expressed as percentage, the obtained measurement uncertainty should be expressed as an integer (number without decimal places), while if measurement uncertainty is expressed as absolute value, it should be expressed in the same way as measured result (as an integer or with the same number of decimal places as measured result). For example, for HbA_1c_ the measurement uncertainty can be expressed as 48 mmol/mol ± 3% or 48 mmol/mol ± 1.44 mmol/mol (≈ 1 mmol/mol). Namely, if the measured result for HbA_1c_ is 48 mmol/mol and the estimated measurement uncertainty is ± 1 mmol/mol (95% CI) then we can assume that true value of HbA1c is between 47 – 49 mmol/mol with 95% probability.

Measurement uncertainties should be estimated at all IQC levels near clinical decision limits.

If appropriate commercial control sample is not available (with values near clinical decision limit), patient control samples could be used. It should be emphasised that in such cases sample stability study must be assessed in order to cover the period in which that kind of control would be used.

Examples of the measurement uncertainty estimation are listed in Appendix 1.

## Supplementary material

Appendix 1. Example 1.

Appendix 1. Example 2.

Appendix 1. Example 3.

Appendix 2.
