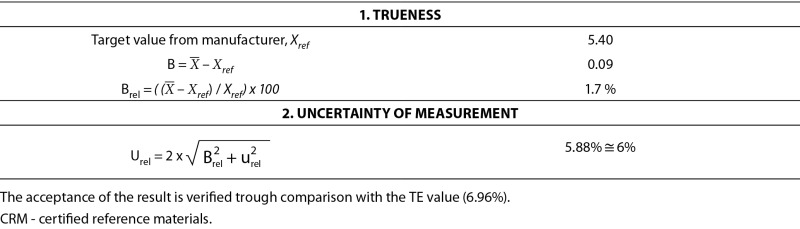# Corrigendum to: Minimum requirements for the estimation of the measurement uncertainty: Recommendations of the joint Working group for uncertainty of measurement of the CSMBLM and CCMB

**DOI:** 10.11613/BM.2018.011201

**Published:** 2017-11-24

**Authors:** Ivana Ćelap, Ines Vukasović, Gordana Juričić, Ana-Maria Šimundić

**Affiliations:** 1 CSMBLM, Committee for the scientific professional development, Working group for uncertainty of measurement of the CSMBLM and CCMB, Croatia; 2 Clinical Institute of Chemistry, University Hospital Centre Sestre milosrdnice, Zagreb, Croatia; 3 Department of Laboratory Diagnostics, General Hospital Pula, Pula, Croatia; 4 Department of Medical Laboratory Diagnostics, Clinical Hospital Sveti Duh, Zagreb, Croatia

This is a correction of *Biochemia Medica* 2017;27(3):030502. DOI: https://doi.org/10.11613/BM.2017.030502.

Since the publication of the article “Minimum requirements for the estimation of the measurement uncertainty: Recommendations of the joint Working group for uncertainty of measurement of the CSMBLM and CCMB”, the authors have noticed that one of the equations presented in Appendix 1 (Example 2) for trueness calculation was published incorrectly. The correct equations for Appendix 1, Example 2 are presented below. The authors apologize for any inconvenience caused to the readers.

## Appendix 1.** Examples of measurement uncertainty estimation**

Example 2. Measurement uncertainty estimation including bias (if CRM is used)