# Supplementary figures and images for: Minimum requirements for the estimation of measurement uncertainty: Recommendations of the joint Working group for uncertainty of measurement of the CSMBLM and CCMB
Source: Biochem Med (Zagreb). 2017 Oct 15;27(3):030502. doi: 10.11613/BM.2017.030502 (PMC5696748; doi:10.11613/BM.2017.030502)

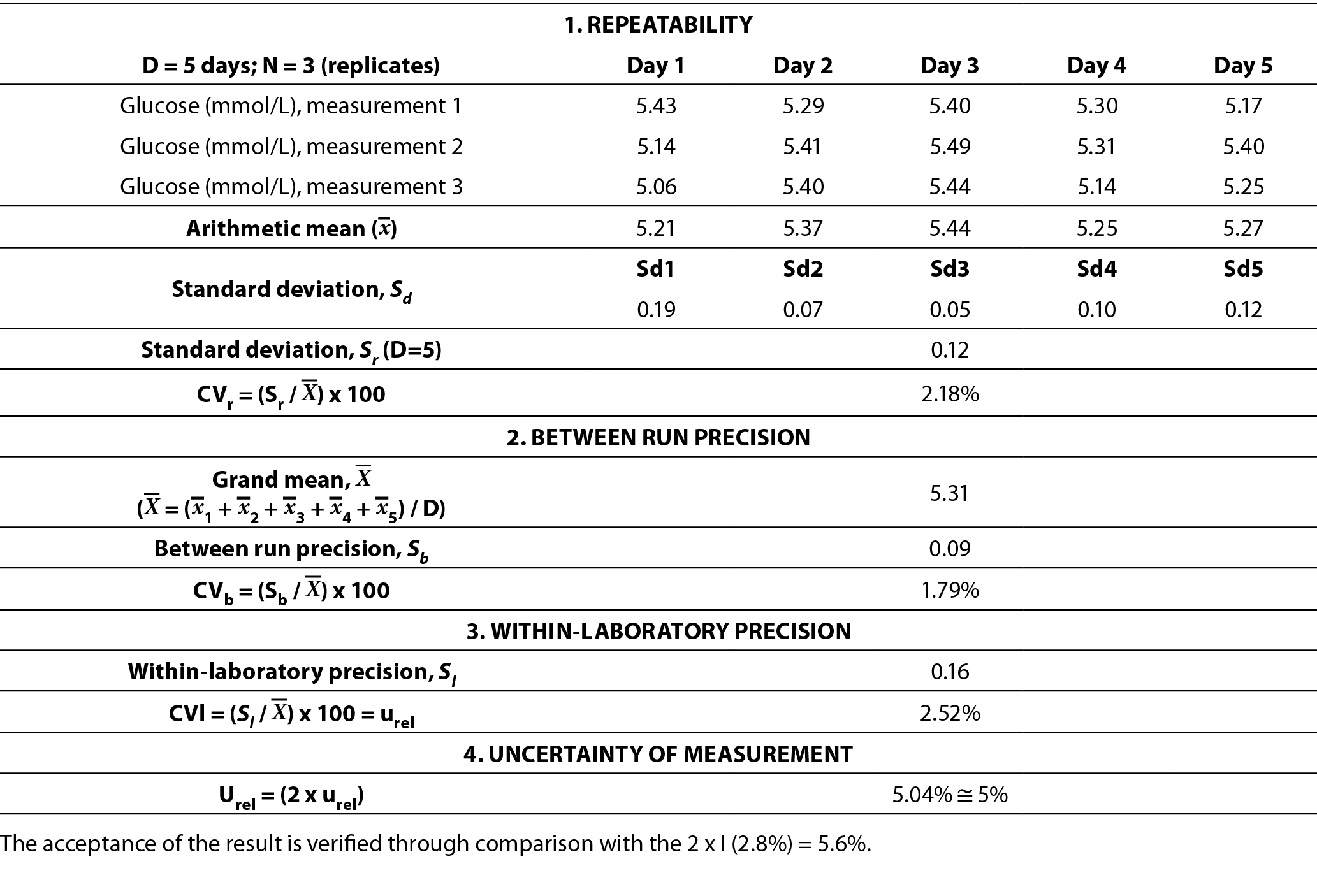

Supplement: Supplementary file 1 — Appendix 1. Example 1. [file bm-27-3-030502-S1.tif]

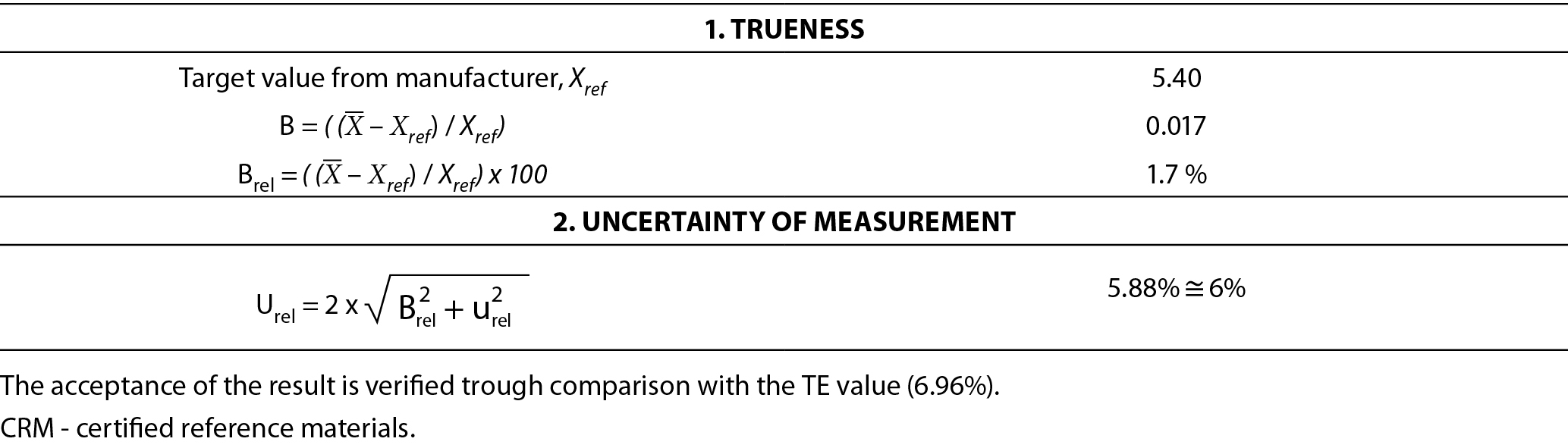

Supplement: Supplementary file 2 — Appendix 1. Example 2. [file bm-27-3-030502-S2.tif]

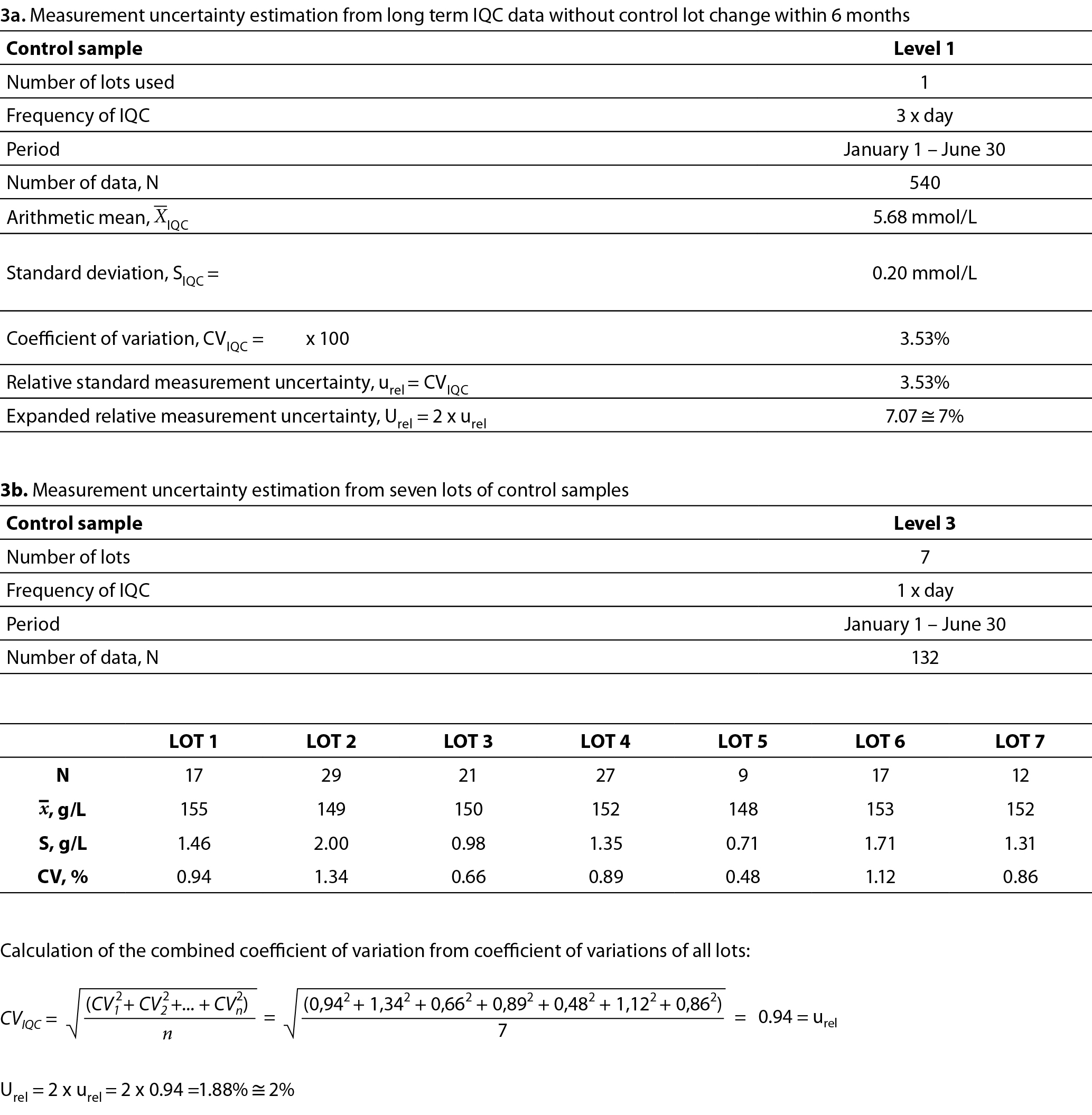

Supplement: Supplementary file 3 — Appendix 1. Example 3. [file bm-27-3-030502-S3.tif]

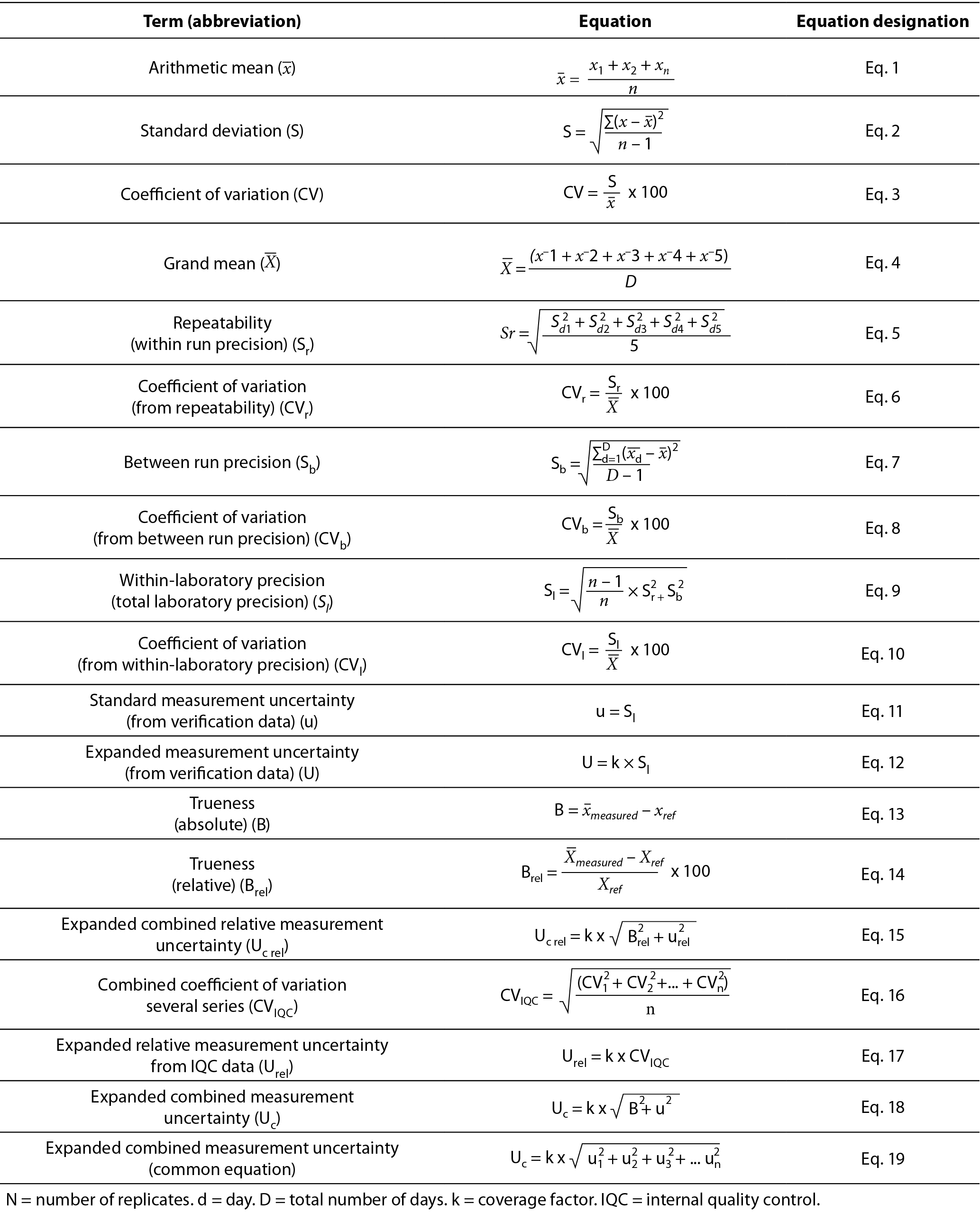

Supplement: Supplementary file 4 — Appendix 2. [file bm-27-3-030502-S4.tif]
